# The Business Problems of a Profession

**Published:** 1911-07-15

**Authors:** 


### BIBLIOGRAPHY.

The Business Problems of a Profession.—This is a little book written by Dr. Frederick Crosby Brush, of New York City, on the business problems connected with the conduct of a practice. It is compiled from the articles contributed by Dr. Brush to the *Dental Digest* on this subject during the past year. In endeavoring to present this subject to the professional attitude there is liable to be the criticism that too much commercialism is manifested; but Dr. Brush has gone as far as possible into ordinary business methods without forgetting that he is writing to professional men. He has presented, of course, only the commercial side of the subject, but he has done this in such a way as not to arouse the antagonism of the ultra professional. It is a very conservative little book intended for the beginner, but many older practitioners would be benefited by a careful adjustment of some of their business methods to Dr. Brush's ideas. It is published by the *Dental Digest*, New York.

